# Knowledge, attitudes and practices of cervical cancer screening among female students enrolled in higher education institutions in Cabo Verde

**DOI:** 10.3332/ecancer.2024.1689

**Published:** 2024-03-28

**Authors:** Natalina Sousa Silva Rocha, Bicho M Clara, Lima Mendonça M Luz, Maria do Rosário Oliveira Martins

**Affiliations:** 1 Faculdade de Ciências e Tecnologias (FCT), Universidade de Cabo Verde, Campus do Palmarejo Grande, CP 7943-010 Praia, Santiago, Cabo Verde; 2 Instituto de Saúde Ambiental (ISAMB), Faculdade de Medicina (FMUL), Universidade de Lisboa, Lisboa 1649-008, Portugal; 3 Instituto Nacional de Saúde Publica, Cabo Verde; 4 GTHM, Instituto de Higiene e Medicina Tropical, Universidade Nova de Lisboa, Lisboa 1349-008, Portugal

**Keywords:** KAP, cervical cancer (CC), screening

## Abstract

Cervical cancer (CC) is the fourth most common cancer among women in the world and the second in Africa. CC is the third most frequent cancer and the first cause of cancer-related death among women in Cabo Verde, with an estimated incidence of 17 cases per 100,000 women. The mortality rate is around 10.5% and represents one of the largest percentages of all types of cancer diagnosed in women in the country. The high mortality rate can be explained by the absence of a national screening programme and a lack of human and physical resources. This study aims to determine the level of knowledge, attitudes and practices on CC among undergraduate female students in Cabo Verde in 2020.

**Methods:**

A descriptive cross-sectional study, using a self-administered structured questionnaire, was conducted in six higher education institutions (HEI) in Cabo Verde between November and December 2020. A total of 618 female undergraduate students were recruited using a simple random sampling technique. Descriptive statistical data analysis was used to report the results.

**Results:**

The response rate was 96.6% (*n* = 618). Most of the participants, 90.6% (549), were single, with average age of 21.79 years (SD =±4). Although most of the participants had already heard about CC (94.6%), most students showed a low knowledge about this disease (86.2%). Moreover, only 9.1% reported having been screened for CC.

**Conclusion:**

Most undergraduate female students enrolled in HEI in Cabo Verde have poor knowledge and unfavourable attitudes toward CC. The level of knowledge is quite unsatisfactory. Within this context, the implementation of health policies focused on human papillomavirus education, prevention strategies, and CC screening is crucial.

## Introduction

Cervical cancer (CC) is the fourth most common cancer among females in the world, after breast, colorectal and lung cancer, representing 6.6% of all female cancer [1, 2]. It is estimated that in the year 2020, the number of new cases of CC diagnosed was 604,127 resulting in approximately 342,000 deaths from such type of cancer, being approximately 90% in low and middle-income countries [1–4]. Africa is the most affected region, with the highest rate incidence in young women, due to poor access to prevention, screening and treatment service. Projections points to 443,000 deaths of CC by 2030, 98% in middle- and low-income countries [3, 4]. CC is the second most common cancer among women in Africa, posing a substantial threat to public health [5].

According to the Cabo Verde CC profile 2020, CC is the third leading cause of female cancer and first most common female cancer diagnosed among women between 15 and 44 years of age, with an estimated incidence of 17% per 100,000 women [6]. CC is the first cause of death from cancer among adult women and the first cause of evacuations for treatment abroad [6, 7]. The mortality rate is around 10.5% and represents one of the largest slices of all types of cancer diagnosed as well as the first cause of death in women in the country [6] Despite existing guidelines for early symptom detection at the primary care level, there is no information on preventive behaviours against CC in Cape Verde. By 2020, national guidelines for the control of CC had not yet implemented [7, 13].

Human papillomavirus (HPV) is the highest significant risk factor linked to CC, a prevalent sexually transmitted infection among young people. HPV16 and HPV18 are responsible for 70% of CC cases [8–10]. Other risk factors such as early sexual activity, long-term contraceptive use, multiple sexual partners, and a history of sexually transmitted infections increase the risk for CC [11, 12]. According to the centres for disease control and prevention, to prevent CC, it is important for women to be vaccinated when eligible, and to submit to regular screening tests. Regular cytology, especially through Pap smears in organised screening programs for high-risk groups, significantly reduces the incidence and mortality rates of invasive CC, particularly in developing countries [14–16].

Studies on the knowledge, attitudes and practices (KAP) of CC in the female population have been implemented in several countries, notably in Africa. For example, a study conducted in Ethiopia with 595 women between the ages of 15 and 49 revealed that about 50% of the participants had a poor knowledge of CC [17]. A similar conclusion was drawn from a study with 514 women in Zimbabwe, where only 20% of the women had heard of CC screening [18]. A study conducted in Mozambique, with students from the ISCISA (Higher Institute of Health Sciences) in Nampula, showed that although 94.4% of the students were aware of the disease, 47.7% were unaware of its transmission [19].

There are no published studies in the country regarding the level of knowledge concerning the risk factors associated with CC and the HPV burden in the population. Based on data from Western Africa, where Cabo Verde is situated, the prevalence of HPV in the general population is estimated at 4.3%. Additionally, 55.7% of CCs are attributed to HPV16 and 18 [13].

HPV programmes were only introduced in the national vaccination program in 2021, covering initially only 10-year-old girls [7,13]. The absence of a structured cytology-based screening program, and adequate health infrastructure, along with the lack of human and physical resources, explain the high number of CC diagnoses and deaths in the country [7].

Despite the growing number of cases of CC in women in Cabo Verde, many of them diagnosed at an advanced stage of the disease. Parte superior do formulárioInsufficient awareness regarding the disease, its risk factors, symptoms, and preventive measures may impact screening practices and the adoption of preventive behaviours against CC. CC primarily impacts women in their younger years, a demographic that often encompasses university students, hence making it pertinent to examine their understanding of this illness [15].

Assessing women’s knowledge about CC is fundamental to define effective intervention programs and health policies directed at CC prevention in Cabo Verde. Unfortunately, there is a lack of data regarding the knowledge of CC in Cape Verde.

The present study aims to explore the KAP on CC screening among female students enrolled in higher education institutions (HEI) in Cabo Verde.

## Method

### Study design

A cross-sectional study was conducted to assess the KAP on CC among higher education female students in Cabo Verde.

Cabo Verde is a sub-Saharan archipelago composed of ten islands and five islets, located in the Atlantic Ocean, approximately 500 km off the western coast of Africa. Cabo Verde is one of the five Portuguese-speaking countries in the continent and has an estimated population of 576,525, out of which 196,716 are females aged 15 years and older [7]. The study was conducted among regular female students enrolled in six HEI in Cabo Verde, namely University of Cabo Verde (UNICV), Jean Piaget University (Unipiaget), Institute of Economic and Social Sciences, University of Mindelo, Institute of Social and Legal Sciences and University of Santiago.

A two-stage sampling technique was used to select the students. First, the total sample size was distributed proportionally to the size of the students at each HEI. Subsequently, one classroom per institution was randomly selected as a starting point. Then systematic random sampling was used to select the remaining classrooms where the data would be collected. As the classrooms were already numbered, there was no need for a reference system for selection.

### Sample size calculation

According to data provided by the Cabo Verdean Ministry of Education, in the 2020–2021 school year, 5,112 female students were enrolled in nine HEI. The prevalence of knowledge of CC (p) in Cabo Verde is unknown. The minimum sample size was calculated based on the following formula [20]:


$$n\;=\;\frac{{\left(\frac{Z_{\alpha }}{2}\right)}^{2}p(1-p)}{d^{2}}$$

where *n* is the expected sample size; *Z* (α/2) is the normal distribution value; *p* is the proportion of CC prevalence assumption (*p* = 50%); *d* is the sampling margin of error (*d* = 0.05). We assume that we have 95% confidence and 5% precision. The final sample size was 768 (384 female students for each island). The value of *p* was set at 50% for sample size calculation.

### Instrument for data collection

The data were collected by using a self-administrated questionnaire adopted from the CC-Knowledge-Prevention-64 questionnaire already translated into Portuguese language [21].

The questionnaire consists of 92 questions, separated into 3 blocks: Sociodemographic characteristics, KAP on CC and Health beliefs related to CC.

- Sociodemographic characteristics include age, course, frequency level, marital status, reproductive characteristics (number of children and sexual partners). We also considered these characteristics, alcohol consumption and tobacco use.
- KAP on CC explore relational issues, risk factors, prevention, symptoms, diagnosis, vaccination, HPV diagnostics, screening of CC, and sexual behaviour of female students.
- Health beliefs related to CC explore the students’ beliefs on CC. These were measured using a five-point Likert scale statements on a scale as follows: strongly agree (SA), agree (A), uncertain (U), disagree (D), strongly disagreed (SD).

### Validity and reliability of instrument

The internal consistency was determined through a test with Cronbach’s α = 0.75.

### Data collection

A field manual was developed by the researcher. Data collectors underwent a 3-day training course to streamline the data collection process. A pre-test was conducted in a pilot study with 20 female students from the UNICV. Data were collected between November and December 2020 using a self-administrated, anonymous questionnaire before participants gave written consent. To maintain anonymity only the age of each student had to be recorded. Questionnaires were distributed to students after describing the purpose of the study, they were completed immediately and collected after the completion of the interviews at the end.

### Data analysis

The data were introduced and analysed using SPSS for Windows v.27. Descriptive analysis was used to describe the KAP about CC. The results were presented using percentages, frequency, tables and graphs.

### Ethical consideration

Ethical approval for this study was obtained from the Health National Research Ethics Committee of Cabo Verde by Resolution reference: nº10/2020. Permission to conduct the study in the Cabo Verde’s HEI was obtained from the Dean of those institutions. All participants gave written consent. Participation in this study was voluntary. All ethical principles of the Helsinki Declaration were followed.

### Outcome measurement

#### Knowledge about CC

The variable ‘level of knowledge about CC’ was calculated using the scores of 14 questions related to risk factor, prevention and symptoms. Each correct response was assigned a one-unit score and zero units for each incorrect response. The scores were added and converted into a percentage. The respondents whose score was 60% or more were considered as ‘having good knowledge’ whereas the respondents whose score was less than 60% were considered as ‘having little knowledge’.

#### Attitudes towards CC

For the construction of the variable attitude about CC, the scores were calculated based upon four questions related to the frequency and reasons for conducting family planning consultation, adding the scores, and converting into a percentage. The respondents whose score was 60% or more were considered ‘as having a positive attitude towards CC’ whereas the respondents whose score was less than 60% ‘where considered ‘as having a negative attitude towards CC’.

#### Practice regarding CC screening

The variable ‘practice relation to CC’ was calculated based on a single issue relating to CC screening. Respondents who had already undergone CC screening were considered as having good practice in relation to CC.

## Results

### Sociodemographic characteristics of the respondents

A total of 640 questionnaires were distributed to female students, with a response rate of 96.6% (*n* = 618). Around 67% (412) were collected on Santiago Island and 33% (206) on S. Vicente Island.

As shown in Table 1, 90.6% (549) of the respondents were single, 7.4% (46) cohabited with a partner, and 1.5% (9) were married. The average age was 22 years and 43.4% (268) were aged between 17 and 20 years. Almost half 44.6% (275) of the students participating were enrolled in the first year and the highest percentage 27.2% (168) are from the UNICV.

### Participants’ knowledge about CC

Most (86.2%) of female students enrolled in HEI in Cabo Verde have little knowledge about CC and a negative attitude (67.3%) toward CC (Table 2). Almost all respondents, 96.4% (591), had heard of CC and almost three-quarters 71% (428) of them noted that it is easy to obtain information about CC.

More than half of them, 388 (63.4%), did not know what causes CC. Almost a quarter, 26.5% (162) of the students knew that it is caused by a virus and these 64.2% (162) correctly identified the HPV as the virus responsible for the disease. Similarly, almost three quarters, 70.3% (428) of the participants didn’t know the answers when asked about HPV transmission.

Most female students’ participating in this study correctly identified multiple partners 63.3% (329) and family history 61.7% (321) as risk factors for CC, and 12.3% (76) did not know the answer. However, a few participants 17.7% (92) correctly selected smoking habits as a risk factor for CC.

**Figure 1.**
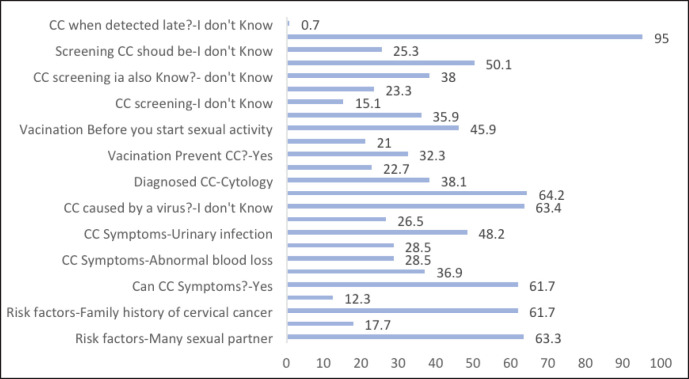
Percentage of student answering correctly to the question or that don’t know.

More than half of the participants, 61.7% (373), knew about symptoms of CC and 37% (223) didn’t know about them. Out of the 373 participants who said that CC has symptoms, almost three-quarters of them, 72.9% (269), correctly identified abnormal blood loss as symptoms of the disease, and almost half of them, 49.1% (173), reported that urinary tract infection is a symptom of CC (Figure 1).

Vaccination is acknowledged as a means of preventing CC by almost a third, 32.3% (184), of respondents in this survey. Pap smears, which should be done after the beginning of sexual activity (20.9%), were identified by almost a quarter, 23.3% (109), as a screening method to prevent the disease. The majority 95% (530) of those surveyed are aware that CC, if diagnosed late, can lead to death.

### Participants’ attitudes and practice towards CC

Table 3 shows that less than a third, 32.7% (202), of the participants have a good attitude towards CC and 67.3% (416) have a poor attitude towards CC. The majority, 95% (530), are aware that CC, if diagnosed late, can lead to death, but more than half, 52.9% (321), do not attend family planning consultations at health centres because they don’t feel the need to do it (66.9%, *n* = 180), Table 2.

Table 4 shows that nearly all participants, 83% (508), have had sexual experience and 41% (213) at the age of ≤17 years. Only 9.1% (55) of them had been screened for CC and almost half, 47.5% (249), of the participants in this study reported not using condoms in their last relationship.

### Beliefs about CC

Table 5 reveals that almost 70% of the respondents disagree that they are more likely to develop CC than other women (68.8%, *n* = 413) and likely (and that they are likely) to develop CC in the next 5 years.

More than half of the participants agree that cytology (pap smears) is the best way to detect cervical lesions (58.0%, *n* = 344) and performing cytology entails a fear of finding something abnormal in the results (60.0%, *n* = 356). Almost half of them agree that a Pap smear will decrease the chances of dying from CC (48.3%, *n* = 288). A large proportion of participants disagree or are undecided to say that cytology is expensive (65.7%, *n* = 390), embarrassing (72.6%, *n* = 428) and painful (77.0%, *n* = 456).

Almost 60% of the respondents disagree that it is irrelevant to think about having a cytology test (58.0, *n* = 344) and less than half (43.7%, *n* = 261) that they do not remember to schedule it.

## Discussion

CCr is the most important cause of death among women in Cabo Verde particularly those aged 15–44. HPV is a common sexually transmitted virus, associated with 99% of cases of CC. In their lifetime, sexually active women will be infected at least once [21-23]. In 2020, Cabo Verde had not yet implemented national guidelines for the management of CC. Knowing the symptoms, risk factors and prevention of CC is crucial to reduce mortality among women in Cabo Verde. This study was conducted to assess the KAP of CC among female students in HEI in Cabo Verde. It took place between November and December 2020, and it is the first study to measure KAP about CC in this country.

Research has shown that the majority (95.3%) of the participants have heard of CC but more than half, 388 (63.4%), didn’t know the causal agent of CC. Out of those who had heard about CC, nearly 71,4%, got the information in the mass media. This result is consistent with, Mapanga *et al* [25] KAP of young people in Zimbabwe on CC and HPV, current screening methods and vaccination. The authors reported that majority 656 (87.7%) students with secondary and university degree have heard about CC disease and 53% of them didn’t know the causative agent of CC. Additionally, Getaneh *et al* [26] examined KAP on CC screening among undergraduate female students in the University of Gondar (Ethiopia) and this study revealed that majority 363 (90%) of their participants respondents have heard about CC, whilst 42.4% did not know the causative agent of the disease.

This finding may be due to students recognising CC as a public health issue, but not knowing what causes it. Understanding the disease itself does not always ensure a thorough understanding of the risk factors associated with it. Students’ limited knowledge of the disease may explain the findings of this study.

Students say they are familiar with CC, probably due to exposure in the media, increased accessibility to smartphones or awareness campaigns at universities. However, they often do not understand the underlying causes of the disease. This deficiency may be related to their limited involvement with health professionals and the general lack of awareness of the disease, on the part of both health professionals and relevant organisations.

In addition to HPV, other risk factors are associated with CC, include early sexual intercourse, multiple sexual partners, and a history of sexually transmitted diseases [11, 12]. In our study, female students correctly identified multiple partners as the highest risk factor 329 (63.3%) for developing the disease. The percentage is lower than that reported by Mofolo *et al* [28] among first-year female students in residences at the University of the Free State in South Africa. In their study, 80.4% of the respondents identified multiple sexual partners as a risk factor for CC. On the contrary, it surpasses the percentage found by Tadesse *et al* [29] among female students at the University of Sciences and Technologies in Ethiopia, which was 40.5%. Getaneh *et al* [26] discovered that only 25% of female students recognised multiple partners as a risk factor for CC.

CC is a significant health problem in Africa, where the burden of the disease is substantial due to various factors, such as limited access to screening services and a lack of awareness of risk factors. The percentage of knowledge about CC in Africa varies according to the countries and regions of the continent. In some areas, there may be higher levels of awareness due to specific educational campaigns, access to health services, and partnerships with international organisations, which could justify these results.

CCr is characterised by pelvic pain, abnormal vaginal bleeding, vaginal bleeding between menstrual periods, and pain during sexual intercourse [32]. Also, a total of 373 students stated that they know CC symptoms but only 28.5% of them identified irregular vaginal bleeding as one of such symptoms. This result is in accordance with Binka *et al* [30], in a study among female undergraduate students in Ghana, in which (31.0%) of the participants identified vaginal bleeding as a CC symptom. These results can be explained by the low awareness of the participants as well as the general population about CC risk factors and its associated symptoms.

For Touch and Oh [31], HPV vaccination can be the most effective prevention method, particularly in countries with weak health systems, contributing to a substantial reduction in morbidity and mortality. HPV immunisation in Cabo Verde has been introduced only for 10-year-old girls in April 2021. None of the participants in our study have been vaccinated, although almost a third of them, 184 (32.3%), recognise vaccination as a CC preventive measure.

According to the WHO, population-based screening programmes reduce in 80% the incidence of CC in women aged 21–64. In the study, almost a quarter, 109 (23.3%), of the respondents identified the pap test as a screening method to prevent the disease but, surprisingly, only 9% of the participants had undergone CC screening. This result can be explained by the low level of knowledge about CC among the students participating in this study. More and better dissemination, sensitisation, and information campaigns on CC are needed.

Only 13.8% (*n* = 85) of the participants had a good knowledge of CC. This finding suggests that HEI students need to acquire solid knowledge about the disease that will encourage preventive actions. This percentage is lower than those found in similar studies conducted in other countries in Africa, namely a study held among students at the University of Buea in Cameroon by Halle-Ekane *et al* [5]. As little as 32.7% of the participants in this study had a positive attitude toward CC in Cabo Verde, which is in line with the results reported by Usman *et al* [33] (34,2%) in Uganda, and lower than those described by Getaneh *et al* [26] (67.7%), University of Northwest Ethiopia.

Our results show that although students claim to be familiar with CC, there’s a clear lack of understanding about its cause and transmission, which can have a profound impact on women’s health in Cape Verde. The dependance on the media for CC information reflect trends seen in studies conducted in other countries, suggesting a common pattern in how students acquire knowledge about the disease [25, 5, 35]. In addition, variations in the identification of risk factors and differences in symptom recognition across studies highlight significant gaps in student awareness that require immediate attention.

Despite some awareness of screening methods, there is a low uptake of cervical screening among students, indicating a gap between knowledge and action. The results also show a significant lack of comprehensive knowledge and less positive attitudes towards CC among Cape Verdean students compared to students in other African countries [16, 5, 24, 34]. This highlights the urgent need for tailored educational interventions to raise awareness and promote preventive measures among university students in Cape Verde.

## Limitations of the study

This study was conducted during the COVID-19 pandemic. In the period of data collection, higher education students enrolled in previous years were attending classes in eLearning mode. Such a context can have bias our results towards younger students. In some institutions it was not possible to achieve the required number of questionnaires.

## Conclusion

While nearly all participants are familiar with CC, they are unaware of the risk factors and symptoms of CC. This study revealed that female students in HEI in Cabo Verde have poor knowledge of CC and very poor screening behaviour for CC. It draws attention to the need to implement campaigns among students regarding the risk factors, symptoms, and preventive measures associated with CC. Formulating health policies aimed at promoting awareness and preventing CC in Cabo Verde is of paramount importance to reduce the burden of this disease in the country.

## Conflicts of interest

The authors declare that they have no competing interests.

## Funding

This study was funded by the Cabo Verde National Health Institute (INSP) and by GHTM, IHMT, Universidade Nova de Lisboa.

## Figures and Tables

**Table 1.** Sociodemographic and reproductive characteristics of female students in higher education institutions, Cabo Verde year 2020.

Respondents (N) = 618			
Variables	Category	Frequency	Percentage (%)
Age	17-20	268	43.4
	21-24	233	37.7
	25-28	63	10.2
	>28	54	8.7
Marital status	Single	549	90.6
	Married	9	1.5
	Live with partners	46	7.6
Children	Yes	147	35.7
	No	426	74.3
Scholl year	I	275	46.6
	II	103	17.5
	III	127	21.5
	IV	85	14.4
Higher education institutions	University of Cabo Verde	168	27.2
	University Jean Piajet	108	17.5
	University of Santiago	97	15.7
	University of Mindelo	79	12.8
	Higher Institute of Social and Economic Sciences	109	17.6
	Higher Institute of Social and Legal Sciences	57	9.2

**Table 2.** Attitudes towards CC among female students in higher education institution in Cabo Verde.

Variable	Category	Frequency	Percentage (%)
Whenever you notice a vaginal infection, what do you do?	Looking for answers in natural medicine	34	6.2
	Wait for the infection to resolve spontaneously	56	10.2
	Refer to a health care professional	439	79.7
	Go to the pharmacy	22	4.0
Attends family planning consultation at the health center/ gynecology?	Yes	286	47.1
	No	321	52.9
If yes, what is the reason?	Routine consultation / surveillance	259	89.0
	Consultation for illness	32	11.0
If no, what is the reason?	For not feeling the need	180	66.9
	For not feeling comfortable	78	29.0
	Because the doctor is a men	11	4.1

**Table 3.** Level of knowledge attitudes and practices of CC among female students in higher education institution in Cabo Verde

Variable	Category	Frequency	Percentage (%)
Level of Knowledge	Good Knowledge	85	13.8
	Little Knowledge	533	86.2
Level of Attitude	Negative attitude	416	67.3
	Positive attitude	202	32.7
Practices	Good practice	55	9.1
	Poor practice	531	88.2

**Table 4.** Practices regarding CC among female students in higher education institution in Cabo Verde

Variable	Category	Frequency	Percentage (%)
Sexual experience	Yes	508	83.0
	No	83	11.9
	Didn´t answer	31	5.1
Age of the first partner	≤ 17	213	41
	>17	316	59.0
Have you ever cervical cancer screening?	Yes	55	9.1
	No	531	88.2
If so, how often?	Once a year	29	59.2
	Two in two years	13	26.5
	Three in three years	4	8.2
	From five to ten years	3	6.1
Number of sexual partners (n=108)	Single	60	55.6
	Multiple	47	43.5

**Table 5.** Beliefs about Cervical Cancer among female students in higher education institution in Cabo Verde

Variable	Strongly disagree	Disagree	Undecided	Agree	Strongly agree
Do you think it is likely that you could get cervical cancer?	82(13.7)	154(25.7)	172(28.7)	173(28.6)	19(3.2)
Do you think that the probability of developing cervical cancer in the next few years is high?	103(17.1)	227(37.6)	167(27.6)	82(13.6)	26(4.1)
Do you feel that throughout your life you could have cervical cancer?	92(15.5)	203(34.2)	197(33.2)	92(15.5)	10(1.7)
Do you think you are more likely to get cervical cancer than most women?	176(29.3)	237(39.5)	118(19.7)	50(8.3)	19(3.2)
Is the development of cervical cancer something you may be experiencing right now?	145(24.4)	188(31.6)	145(24.4)	83(13.9)	34(5.7)
Are you worried that you might develop cervical cancer in the near future?	61(10.0)	91(15.0)	97(16.0)	237(39.0)	121(19.9)
Does thinking about cervical cancer scare you?	43(7.1)	64(10.6)	45(7.5)	223(37.0)	227(37.7)
When you think about cervical cancer, does your heartbeat faster?	72(11.8)	105(25.1)	90(14.9)	177(29.3)	108(17.9)
Are you afraid to think about cervical cancer?	78(12.9)	152(25.1)	90(14.9)	177(29.3)	108(17.9)
Do you think that if you had cervical cancer the problems you would have would last a long time?	47(7.8)	142(23.7)	181(30.2)	161(26.8)	68(11.3)
Do you think if you had cervical cancer your whole life would change?	44(7.4)	70(11.7)	87(14.5)	207(34.6)	190(31.8)
If I developed cervical cancer, wouldn't I live for more than 5 years?	108(18.2)	175(29.4)	221(37.1)	60(10.1)	31(5.2)
Does cytology help detect uterine lesions earlier?	24(4.0)	42(7.1)	129(21.7)	236(39.7)	163(27.4)
If the cytology shows a lesion in the uterus, won't the cancer treatment be so bad?	51(8.6)	82(13.8)	206(34.6)	192(32.2)	64(10.8)
Is cytology the best way to detect small lesions in the uterus?	29(4.9)	40(6.7)	180(30.4)	228(38.4)	116(19.6)
Will having a cytology lower your chances of dying from cervical cancer?	55(9.2)	72(12.1)	181(30.4)	191(32.0)	97(16.3)
When you have a cytology test, are you afraid of finding something wrong?	42(7.1)	81(10.3)	134(22.6)	227(38.2)	129(21.8)
Are you afraid of having a cytology test because you don't understand how it is done?	97(16.2)	162(27.1)	121(20.3)	149(24.9)	69(11.5)
Would a cytology test be embarrassing?	70(11.9)	139(23.6)	219(37.1)	124(21.0)	38(6.4)
Would it take a long time to do a cytological test?	70(12.1)	145(25.1)	277(47.9)	56(9.7)	30(5.2)
Would a cytology test be painful?	70(11.8)	121(20.4)	265(44.8)	97(16.4)	39(6.6)
Would having a cytology test force me to miss class?	103(17.5)	136(23.1)	207(35.2)	106(18.0)	36(6.1)
Do you have other problems more important than thinking about doing a cytology?	154(26.0)	190(32.0)	145(24.4)	71(12.0)	33(5.6)
Would it be expensive to do a cytology test?	74(12.5)	104(17.5)	212(35.7)	145(24.4)	59(9.9)
I can't remember to schedule the exam (cytology)	121(20.5)	137(23.2)	171(29.0)	105(17.8)	56(9.5)
